# Loss of Midbrain Dopamine Neurons Does Not Alter GABAergic Inhibition Mediated by Parvalbumin-Expressing Interneurons in Mouse Primary Motor Cortex

**DOI:** 10.1523/ENEURO.0010-24.2024

**Published:** 2024-05-07

**Authors:** Suraj Cherian, Gabriel Simms, Liqiang Chen, Hong-Yuan Chu

**Affiliations:** Department of Neurodegenerative Science, Van Andel Research Institute, Grand Rapids, Michigan 49503

**Keywords:** 6-OHDA, dopamine, motor cortex, optogenetics, Parkinson's disease, parvalbumin

## Abstract

The primary motor cortex (M1) integrates sensory and cognitive inputs to generate voluntary movement. Its functional impairments have been implicated in the pathophysiology of motor symptoms in Parkinson's disease (PD). Specifically, dopaminergic degeneration and basal ganglia dysfunction entrain M1 neurons into the abnormally synchronized bursting pattern of activity throughout the cortico-basal ganglia-thalamocortical network. However, how degeneration of the midbrain dopaminergic neurons affects the anatomy, microcircuit connectivity, and function of the M1 network remains poorly understood. The present study examined whether and how the loss of dopamine (DA) affects the morphology, cellular excitability, and synaptic physiology of Layer 5 parvalbumin-expressing (PV^+^) cells in the M1 of mice of both sexes. Here, we reported that loss of midbrain dopaminergic neurons does not alter the number, morphology, and physiology of Layer 5 PV^+^ cells in M1. Moreover, we demonstrated that the number of perisomatic PV^+^ puncta of M1 pyramidal neurons as well as their functional innervation of cortical pyramidal neurons were not altered following the loss of DA. Together, the present study documents an intact GABAergic inhibitory network formed by PV^+^ cells following the loss of midbrain dopaminergic neurons.

## Significance Statement

The pyramidal neurons in the motor cortex manifest a highly synchronized bursting pattern of activity in the parkinsonian state, but the underlying circuit mechanisms are poorly understood. One can easily consider the PV interneuron–mediated inhibitory network as a potential microcircuitry mechanism. However, whether loss of DA affects the cortical PV^+^ network remains unknown. The present work documented that loss of DA in the parkinsonian state does not alter the number, morphology, cellular excitability, and synaptic physiology of PV^+^ cells in M1. An intact robust PV^+^ perisomatic inhibition of pyramidal neurons provides a microcircuit substrate for thalamic afferents to entrain cortical neurons to pathological oscillations throughout the cortico-basal ganglia-thalamocortical network in the parkinsonian state.

## Introduction

Degeneration of dopaminergic neurons in the substantia nigra pars compacta (SNc) is the defining pathology of Parkinson's disease (PD). Decreased dopamine (DA) levels and the associated adaptative neural network changes, particularly in the basal ganglia nuclei, have been closely linked with the pathophysiology of motor symptoms in PD (Galvan and Wichmann, 2008; McGregor and Nelson, 2019). Enhanced bursting pattern of neuronal firing, which is often highly synchronized among neurons within and between brain areas, has been reported in both people with PD and DA-depleted parkinsonian animals (Galvan and Wichmann, 2008). This abnormal pattern of neural activity is closely linked with the motor deficits in PD, particularly the bradykinesia. Evidence from the basal ganglia field suggests that the exaggerated bursting pattern of activity involves maladaptive changes of both cellular properties and synaptic inputs, as well as their interactions in the parkinsonian state (Baufreton et al., 2005; Chu et al., 2015; Mastro et al., 2017; McGregor and Nelson, 2019).[Table T1]

**Table 1. T1:** Physiological properties of PV+ cells in M1 of controls and DD mice

Figure number		Sample size (mice, slices, cells)	Mean±SEM	Median [25–75^th^ percentile]	Statistic methods	U/F value	CI lower limit	CI upper limit	Difference of medians	*p*-value
	M1 TH (ratio % area)	4 mice, 6 sections (control)	1.25 ± 0.38	1.01 [0.56,1.74]	Mann Whitney	*U* = 3	0.512	3.077	−0.6585	0.0017
		5 mice, 10 sections (DD)	0.34 ± 0.08	0.35 [0.097,0.51]			0.055	0.539		
Figure 1*G*	PV cell density ratio (stereology)	5 mice, 10 sections (control)	0.93 ± 0.08	1.02 [0.73,1.08]	Mann Whitney	*U* = 5	0.69	1.1	0.136	0.082
		6 mice, 12 sections (DD)	1.16 ± 0.06	1.16 [1.02,1.25]			0.99	1.4		
	VTA cell density ratio (stereology)	3 mice, 6 sections (control)	1.07 ± 0.14	1.1 [0.82,1.3]	Mann Whitney	*U* = 0				
		3 mice, 6 sections (DD)	0.432 ± 0.02	0.428 [0.398,0.469]						
Figure 2*C*	Soma volume	3 mice, 5 slices, 12 cells (control)	1,291 ± 202	1,022 [728,1,838]	Mann Whitney	*U* = 46	709.1	1,888	−187	0.0868
		3 mice, 6 slices, 13 cells (DD)	820 ± 118	835 [376,1,177]			351.1	1,186		
Figure 2*D*	Dendritic length	3 mice, 5 slices, 12cells (control)	2,138 ± 212	2,151 [1,543,2,856]	Mann Whitney	*U* = 57	1,516	2,965	318.3	0.2701
		3 mice, 6 slices, 13 cells (DD)	2,534 ± 205	2,469 [1,914,2,786]			1,871	2,947		
Figure 2*E*	Primary dendrites	3 mice, 5 slices, 12 cells (control)	6.3 ± 0.5	6 [5,7.8]	Mann Whitney	*U* = 59	5	8	1	0.3165
		3 mice, 6 slices, 13 cells (DD)	6.8 ± 0.3	7 [6,8]			6	8		
Figure 2*F*	Branch points	3 mice, 5 slices, 12 cells (control)	12.7 ± 1.3	13.5 [9,15.8]	Mann Whitney	*U* = 78	8	16	−0.5	> 0.999
		3 mice, 6 slices, 13 cells (DD)	13.6 ± 0.8	13 [12,16]			12	17		
Figure 3*E*	Capacitance (pF)	7 mice, PV-CC, 25 cells (control)	44.13 ± 3.39	40.1 [30.5,52.4]	Two-way ANOVA (Tukeys)	*F*_(3,69)_ = 19.19	37	49.4	—	0.0082
		8 mice, PV-CC, 26 cells (DD)	34.39 ± 2.29	30.9 [27.7,40.9]			28.2	37.98		
		7 mice, PV-IN, 21 cells (control)	24.5 ± 1.7	24.1 [19.5,28.2]			19.9	28.2		
		8 mice, PV-IN, 31 cells (DD)	24.4 ± 1.42	22.7 [18.7,28.3]			19.45	26.31		
Figure 3*F*	Input resistance (MΩ)	7 mice, PV-CC, 25 cells (control)	58.23 ± 3.14	54.8 [44,72]	Two-way ANOVA (Tukeys)	*F*_(3,69)_ = 13.21	44.7	65.6	—	> 0.05
		8 mice, PV-CC, 26 cells (DD)	61.7 ± 3.5	56.2 [51.4,70]			53	65.8		
		7 mice, PV-IN, 21 cells (control)	93.6 ± 7.12	85.2 [75.3,98.6]			75.5	97.6		
		8 mice, PV-IN, 31 cells (DD)	79.1 ± 3.3	74.7 [68.5,92.8]			69.5	82.8		
Figure 3*G*	Rheobase (pA)	7 mice, PV-CC, 25 cells (control)	400.2 ± 29.8	415 [250,488]	Two-way ANOVA (Tukeys)	*F*_(3,69)_ = 33.5	302	462	—	> 0.05
		8 mice, PV-CC, 26 cells (DD)	354.1 ± 20.8	350 [278,431]			290	400		
		7 mice, PV-IN, 21 cells (control)	170.8 ± 18.7	151 [90,228]			90	221		
		8 mice, PV-IN, 31 cells (DD)	162.2 ± 9.97	150 [120,200]			144	195		
Figure3*H*	AP threshold (mV)	7 mice, PV-CC, 25 cells (control)	−42.6 ± 1.01	−41.9 [−45.4,−39.9]	Two-way ANOVA (Tukeys)	*F*_(3,69)_ = 3.856	−43.6	−40.5	—	> 0.05
		8 mice, PV-CC, 26 cells (DD)	−44.4 ± 0.84	−44.8 [−48.3,−41.3]			−47.5	−42.3		
		7 mice, PV-IN, 21 cells (control)	−45.8 ± 1.0	−47 [−49.1,−44.8]			−48.8	−45		
		8 mice, PV-IN, 31 cells (DD)	−47.1 ± 1.1	−46.5 [−48.7,−44.3]			−48.1	−44.9		
Figure3*J*	AP half-width (ms)	7 mice, PV-CC, 25 cells (control)	0.163 ± 0.005	0.16 [0.15,0.18]	Two-way ANOVA (Tukeys)	*F*_(3,69)_ = 10.41	0.149	0.173	—	> 0.05
		8 mice, PV-CC, 26 cells (DD)	0.168 ± 0.003	0.17 [0.15,0.18]			0.16	0.174		
		7 mice, PV-IN, 21 cells (control)	0.228 ± 0.02	0.21 [0.17,0.23]			0.18	0.232		
		8 mice, PV-IN, 31 cells (DD)	0.22 ± 0.01	0.2 [0.19,0.23]			0.19	0.223		
Figure3*K*	AP height (mV)	7 mice, PV-CC, 25 cells (control)	75.93 ± 1.3	76.3 [73,79.2]	Two-way ANOVA (Tukeys)	*F*_(3,69)_ = 20.9	73.74	79	—	> 0.05
		8 mice, PV-CC, 26 cells (DD)	78.89 ± 1.68	80.3 [73.3,85.9]			74.06	85.6		
		7 mice, PV-IN, 21 cells (control)	88.2 ± 1.52	89.1 [82.4,94.2]			83.3	93.6		
		8 mice, PV-IN, 31 cells (DD)	87.8 ± 1.1	88.3 [85.2,92.1]			86.1	91.2		
Figure4*B*	F–I curve (PV-IN)	7 mice, PV-IN, 21 cells (control)	—	—	Two-way ANOVA	*F*_(1,846)_ = 1.128	—	—	—	0.2885
		8 mice, PV-IN, 31 cells (DD)	—	—						
Figure4*D*	F–I curve (PV-CC)	7 mice, PV-CC, 25 cells (control)	—	—	Two-way ANOVA	*F*_(1,829_) = 0.3152	—	—	—	0.5746
		8 mice, PV-CC, 26 cells (DD)	—	—						
Figure5*B*	Perisomatic PV puncta (PT)	4 mice, 39 cells (control)	38.51 ± 2.46	36 [26,50]	Mann Whitney	*U* = 757	28	48	−1	0.28
		4 mice, 45 cells (DD)	33.82 ± 1.93	35 [26,45.5]			30	40		
Figure5*D*	Perisomatic PV puncta (IT)	4 mice, 37 cells (control)	32.2 ± 1.71	33 [24.5,39]	Mann Whitney	*U* = 787.5	30	36	0	0.8
		4 mice, 44 cells (DD)	32.7 ± 1.4	33 [24,39]			29	38		
Figure5*K*	IPSC amplitude vs stimulus intensity (PT)	4 mice, 33 cells (control)	—	—	Two-way ANOVA	*F*_(1,492)_ = 0.4908	—	—	—	0.4839
		8 mice, 51 cells (DD)	—	—			—	—		
Figure5*L*	PPR (PT)	4 mice, 33 cells (control)	0.659 ± 0.027	0.7 [0.58,0.79]	Mann Whitney	*U* = 824	0.606	0.76	0.007	0.9
		8 mice, 51 cells (DD)	0.648 ± 0.023	0.7 [0.53,0.79]			0.617	0.747		
Figure5*M*	IPSC amplitude vs stimulus intensity (IT)	4 mice, 32 cells (control)	—	—	Two-way ANOVA	*F*_(1,373)_ = 5.905	—	—	—	0.0156
		5 mice, 33 cells (DD)	—	—			—	—		
Figure5*N*	PPR (IT)	4 mice, 32 cells (control)	0.64 ± 0.025	0.65 [0.54,0.75]	Mann Whitney	*U* = 436	0.593	0.733	−0.04344	0.2316
		5 mice, 33 cells (DD)	0.59 ± 0.03	0.61 [0.47,0.72]			0.491	0.694		

The primary motor cortex (M1) was proposed to be passively suppressed by the pathological inhibition of the basal ganglia via the motor thalamus in PD, and its hypoactivity is believed to underlie the hypokinetic symptoms in parkinsonism (Albin et al., 1989; DeLong, 1990; Galvan and Wichmann, 2008; McGregor and Nelson, 2019). However, in vivo electrophysiology (Goldberg et al., 2002; Pasquereau and Turner, 2011) and imaging studies (Aeed et al., 2021) in parkinsonian animals reported an exaggerated highly synchronized bursting pattern of activity in the M1, which perhaps limits the information processing capacity of the cortical circuit. To understand the cellular and circuit mechanisms of the abnormal pattern of neuronal firing in parkinsonism, we recently reported that in parkinsonian mice loss of DA neurons selectively impairs the intrinsic excitability of M1 pyramidal tract (PT) neurons and decreases their excitatory inputs arising from the motor thalamus (Chen et al., 2021, 2023). These changes might be part of the biophysical determinants of the abnormal pattern of neuronal firing in M1 but still cannot fully explain the abnormal pattern of M1 PT neurons.

The gamma-aminobutyric acid–containing (GABAergic) neurons comprise ∼20% of the total number of cortical neurons and play a pivotal role in controlling cortical network activity and dynamics. The parvalbumin-expressing (PV^+^) cells represent ∼40% of GABAergic interneurons in rodent neocortex (Tanaka et al., 2011; Naka and Adesnik, 2016; Tremblay et al., 2016), which control the timing of cortical pyramidal neuronal activity through the robust perisomatic inhibition. In the M1, PV^+^ cells contribute to the learning of motor skills (Estebanez et al., 2017). Their activity is modulated by dopaminergic inputs from the ventral tegmental area (VTA) in rodents (Hosp et al., 2011; Cousineau et al., 2022). Altogether, it is plausible to predict that PV^+^ cells and their inhibitory synapses in M1 contribute to the bursting pattern of activity of cortical pyramidal neurons in parkinsonism. However, whether the loss of midbrain dopaminergic neurons affects the morphology and physiology of M1 PV^+^ cells remain undefined.

In the present study, we compared the cellular and synaptic properties of M1 PV^+^ cells of both control and DA-depleted (DD) parkinsonian mice to understand how PV^+^ cells and their synaptic connections may contribute to the abnormal cortical network activity in PD. We found that loss of midbrain DA neurons does not alter the GABAergic network mediated by PV^+^ cells, suggesting that the capacity of this network in gating pyramidal neuronal activity is intact in parkinsonism and that it can be recruited by cortical and subcortical inputs to synchronize M1 pyramidal neuronal firing. These findings provide new insight into our understanding of cortical pathophysiology in the parkinsonian state.

## Materials and Methods

### Animals

All mice were housed up to four animals per cage under a 12 h light/dark cycle with *ad libitum* access to food and water. All animal procedures and experiments were reviewed and approved by the Institutional Animal Care and Use Committee (IACUC) at the Van Andel Institute. Wild-type C57BL/6J mice of both sexes (3–4 months old, RRID:IMSR_JAX:000664) were obtained from the Van Andel Institute vivarium internal colony. Transgenic mouse lines were originally purchased from the Jackson Laboratory. Mouse genotyping was performed by the commercial service of TransnetYX. Homozygous PV-Cre knockin mice (JAX stock no. 017320, RRID: IMSR_JAX:017320) were crossed with Ai14-Rosa26-tdTomato reporter mice (JAX stock no. 007914, RRID:IMSR_JAX:007914) to generate experimental PV-tdTomato reporter mice expressing tdTomato in PV^+^ cells. To study GABAergic synaptic transmission from PV^+^ cells to cortical pyramidal neurons, homozygous PV-Cre knockin mice were crossed with homozygous Ai32-ChR2(H134R-eYFP) mice (JAX stock no. 024109, RRID:IMSR_JAX:024109) to generate experimental PV-ChR2 mice expressing ChR2(H134R)-eYFP in PV^+^ cells and their axon terminals. Mice were randomly assigned to experimental groups.

### Stereotaxic surgeries

Mice were mounted and secured in a stereotaxic frame (David Kopf Instruments) under 2% isoflurane anesthesia. The body temperature of the mouse was maintained by a thermostatic heating pad throughout the procedure. Mice received the following surgeries: (1) 6-hydroxydopamine (6-OHDA, 3–4 µg) injection into the medial forebrain bundle [MFB, from the bregma (in mm), anteroposterior (A-P) = −0.7, mediolateral (M-L) = +1.2, and dorsoventral (D-V) = −4.7 from the brain surface) to induce unilateral DA depletion or vehicle injection into the MFB to serve as vehicle-injected controls; (2) retrobeads (200 nl, 10× dilution) or AAVrg-hsyn-eGFP (RRID:Addgene_50465, 300 nl at 2 × 10^13^ GC/ml) injections into the ipsilateral pontine nuclei [from the bregma (in mm), A-P = −5.0, M-L = +0.6, D-V = −5.0] or contralateral striatum (from the bregma, A-P = +0.2, M-L = −2.3, D-V = −2.9) to retrogradely label PT and intratelencephalic (IT) neurons, respectively. All injections were performed using a 10 µl syringe (Hamilton) mounted on a motorized microinjector (Stoelting) at a speed of 100 nl/min. Details of the stereotaxic injection procedure can be found on Protocols.io (dx.doi.org/10.17504/protocols.io.rm7vzye28lx1/v1).

### Slice preparation for electrophysiology

Three to four weeks after the injections, brain slices were prepared for electrophysiology. Mice were anesthetized with an intraperitoneal injection of avertin (250–300 mg/kg), followed by transcardial perfusion with ice-cold sucrose-based artificial cerebrospinal fluid (ACSF) equilibrated with 95%O_2_/5%CO_2_ and containing (in mM) 230 sucrose, 26 NaHCO_3_, 10 glucose, 10 MgSO_4_, 2.5 KCl, 1.25 NaH_2_PO_4_, 0.5 CaCl_2_, 1 sodium pyruvate, and 0.005 ʟ-glutathione. Coronal brain sections (270 µm) containing the primary motor cortex were prepared using a vibratome (VT1200, Leica Biosystems) in the same sucrose-based solution that was maintained at 4°C using a recirculating chiller (FL300, Julabo). Slices were then held in ACSF equilibrated with 95%O_2_/5%CO_2_, containing (in mM) 126 NaCl, 26 NaHCO_3_, 10 glucose, 2 MgSO_4_, 2.5 KCl, 1.25 NaH_2_PO_4_, 1 sodium pyruvate, and 0.005 ʟ-glutathione at 35°C for 30 min for recovery and then at room temperature until electrophysiology recordings. Details of slice preparation can be found on Protocols.io (dx.doi.org/10.17504/protocols.io.36wgqj2eovk5/v1).

### Brain slice electrophysiology and optogenetics

Brain slices were held in a recording chamber with continuous perfusion (3–4 ml/min) of recording solution containing (in mM) 126 NaCl, 26 NaHCO_3_, 10 glucose, 3 KCl, 1.6 CaCl_2_, 1.5 MgSO_4_, and 1.25 NaH_2_PO_4_. The solution was equilibrated with 95%O_2_/5%CO_2_. All ex vivo brain slice recordings were conducted at 33–34°C using a feedback-controlled in-line heater (TC-324C, Warner Instruments).

Neurons were visualized using a charge-coupled device camera (Retiga Electro, Teledyne Photometrics) and the BOB system (Sutter Instruments) integrated with a BX51 upright microscope and a motorized MP285 micromanipulator (Sutter Instruments). PV^+^ cells and retrogradely labeled pyramidal neurons in Layer 5 were targeted under a 60× water immersion objective lens (Olympus) for whole-cell patch-clamp recording using the dPatch digital patch amplifier system controlled by the SutterPatch software (Sutter Instruments). Data were sampled at 50 KHz and low-pass filtered at 5 KHz (Bessel filter). Borosilicate glass pipettes (outer diameter, 1.5 mm; inner diameter, 0.86 mm; length, 10 cm; item no. BF150-86-10, Sutter Instruments) were pulled by a micropipette puller (P1000, Sutter Instruments; RRID:SCR_021042) and used for patch-clamp recording with a resistance of 3–6 MOhm when measured in extracellular recording solution and filled with the following internal solutions: (1) high-Cl internal solution, containing (in mM) 50 CsCl, 85 cesium methanesulfonate, 3.6 NaCl, 1 MgCl_2_, 10 HEPES, 0.1 Na_4_-EGTA, 2 ATP-Mg, and 0.1 GTP-Na (pH = 7.3, 290 mOsm), or (2) K-gluconate–based internal solution, containing (in mM) 140 K-gluconate, 3.8 NaCl, 1 MgCl_2_, 10 HEPES, 0.1 Na_4_-EGTA, 2 ATP-Mg, and 0.1 GTP-Na (pH = 7.3, 290 mOsm). Resting membrane potential (Vm) was recorded once the whole-cell configuration was obtained. Cellular properties and excitability of PV-INs were studied by injecting a family of current steps ranging from −150 pA to 1 nA in 50 pA increments and with a duration of 1 s. Current injections were from the resting membrane potential (RMP), and no additional holding current was injected. Input resistance (Rm) was determined by measuring the steady-state voltage responses to a series of 1 s hyperpolarizing currents, as the slope of linear fit to the resulting voltage–current relationship. The rheobase was defined as the current intensity to evoke an action potential (AP). AP waveforms at the rheobase were analyzed in detail using Clampfit 11.1 to obtain the following parameters, including the threshold, half-width, and height of AP. Specifically, the AP threshold was determined as the voltage level at which dV/dt exceeded 10 mV/ms. AP height was defined as the voltage difference between the threshold and the peak voltage. AP half-width was measured as the time difference at 50% of AP amplitude. Approximately 10 mV junction potential was not corrected.

Biocytin (0.2%) was added to the K-gluconate–based internal solution to fill PV^+^ cells (∼ 30 min), and the morphology of the filled neuron was revealed using Cy5-conjugated streptavidin. The recordings of the inhibitory postsynaptic currents (IPSCs) arising from PV^+^ cells to cortical pyramidal neurons were conducted in the presence of glutamatergic receptor blockers (i.e., 50 µM D-APV and 20 µM DNQX) under whole-cell voltage-clamp mode (at −60 mV) using the high-Cl^−^ internal solution (see above). Optogenetic stimulation was delivered using a 478 nm light-emitting diode (LED, Sutter Instruments) through a 60× water immersion objective lens (Olympus), centering at the site of patched neurons. The peak amplitude of GABA_A_ receptor–mediated IPSCs was quantified as a measure of synaptic strength. Electrophysiology data were analyzed and quantified using Clampfit 11.1 (Molecular Devices; RRID:SCR_011323).

#### Immunohistochemistry and confocal microscopy

Mice were perfused with ice-cold phosphate-buffered saline (PBS; pH = 7.4) for 5 min and subsequently with 4% PFA mice for 30 min. The brain was then extracted and saved in 4% PFA overnight at 4°C before being resected into 70 µm slices using a VT1000 s vibratome (Leica Biosystems; RRID:SCR_016495) for immunohistochemistry or morphology studies. To quantify striatal tyrosine hydroxylase (TH), brain tissues were fixed in 4% PFA in 0.1 M phosphate buffer overnight at 4°C and then were resectioned into 70 µm sections for immunohistochemistry.

Brain slices were rinsed three times with PBS before being incubated with 0.2% Triton X-100 and 2% normal donkey serum (Sigma-Aldrich) for 60 min at room temperature. Brain slices were then incubated with primary antibodies, including mouse anti-TH (1:2,000; catalog #MAB318, MilliporeSigma; RRID:AB_2201528) or guinea pig anti-PV (1:1,000, catalog #195004, Synaptic Systems) for 48 h at 4°C or overnight at room temperature. Sections were then rinsed three times with PBS and incubated with secondary antibodies for 90 min, including donkey anti-mouse Alexa Fluor 488 (catalog #715-545-150; Jackson ImmunoResearch Laboratories; RRID:AB_2340846), donkey anti-mouse Alexa Fluor 594 (catalog #715-585-150; Jackson ImmunoResearch Laboratories; RRID:AB_2340854), or donkey anti-guinea pig Alexa Fluor 647 (catalog #706-605-148; Jackson ImmunoResearch Laboratories), at room temperature before washing with PBS for three times. To reveal the morphology of biocytin-labeled PV^+^ cells, brain slices were fixed in 4% PFA in 0.1 M phosphate buffer overnight at 4°C, followed by incubation with Cy5-conjugated streptavidin (1:1,000) for 90 min. Brain sections were mounted with VECTASHIELD antifade mounting medium (catalog #H-1000, Vector Laboratories; RRID:AB_2336789) and were cover-slipped and sealed with nail polish.

Immunofluorescence was imaged under 20, 40, or 100× objective lens using a confocal laser scanning microscope (A1R, Nikon; RRID:SCR_020317). TH immunoreactivity (ir) in the striatum of controls and DA-depleted mice was quantified by normalizing the striatal TH-ir from the ipsilateral side to the value from the contralateral hemisphere, with a subtraction of background measured from the cerebral cortex. All mice with 6-OHDA lesions exhibited >80% loss of TH-ir in the ipsilateral striatum compared with the contralateral hemisphere, indicating an almost complete striatal DA depletion. Details of immunohistochemical quantification of TH signals can be found at Protocols.io (dx.doi.org/10. 17504/protocols.io.n2bvj85qngk5/v1). TH-ir in the M1 are more sparse and scattered compared with those in the striatum. To estimate potential changes in the TH-ir axons, a 100× oil immersion objective was used to image TH-ir in Layer 5 of M1. The images were background subtracted followed by thresholding using Otsu’s approach (Otsu, 1979). Particle analysis in Fiji was used to determine TH-ir puncta, and the area covered was determined for each hemisphere. The ratio of area covered by the TH-ir signal from the ipsilateral to the contralateral M1 was calculated and reported (Table 1).

To assess the number of PV^+^ cells in Layer 5 of M1 from both hemispheres using unbiased stereology, confocal images were taken from PFA-fixed sections of PV-tdTomato mice using a 20× oil-immersion objective lens (numerical aperture, 0.75). All confocal images for PV^+^ cell quantification were taken using identical settings (i.e., *x*,*y*, 1,024 × 1024 pixels; digital zoom, 1; 20 µm in depth, 3 µm *z*-step) and analyzed without postprocessing. Stereological quantification of the density of PV-tdTomato cells was conducted by the optical dissector method using Stereo Investigator (MBF Bioscience; West, 1999). Stereological counting of tdTomato-positive (i.e., PV^+^) cells in Layer 5 was counted by an experimenter blinded to treatment groups. Specifically, each brain sections were counted using a grid size of 240 × 240 µm and a counting frame (100 × 100 µm). Dissector height was set to 18 µm with a guard zone of 1 µm. Schaeffer’s coefficient of error was between 10 and 14%. The ratio of cell numbers in the ipsilateral to contralateral M1 was calculated and reported to minimize variabilities between animals due to technical issues. For unbiased stereology analysis of VTA DA neurons, confocal images were taken using 20× oil immersion objective using identical settings (1,024 × 1,024 pixels; 20 µm in depth; 1 µm *z*-step). VTA DA neurons were outlined by TH-ir signals. Sections spaced at 70 µm sections apart were counted using a grid size of 150 × 150 µm and a counting frame (100 × 100 µm). Dissector height and guard zones were used the same as in PV^+^ cell count. The ratio of the number of cells in the ipsilateral to contralateral VTA was reported (Table 1).

Biocytin-labeled PV^+^ cells were imaged using confocal microscopy (A1R, Nikon) under a 40× objective lens (1 µm *z*-step), and the morphology of biocytin-labeled neurons was reconstructed and quantified using the Imaris software (version 9.3; RRID:SCR_007370). eGFP-labeled cortical pyramidal neurons and PV-ir perisomatic puncta were imaged from the top to bottom surfaces using confocal microscopy under a 100× objective lens (0.5 µm step). A researcher blinded to the treatment groups counted the entire number of PV-ir puncta around the eGFP-labeled neurons in the *Z*-stack images using Fiji software (Schindelin et al., 2012).

#### Experimental design and statistical analysis

##### Experimental design

The purpose of the present study was to determine whether the cellular and synaptic properties of PV^+^ cells in the M1 were affected by DA depletion in parkinsonian mice. Both male and female adult mice were used and assigned to different treatment groups randomly. Neurotoxin 6-OHDA was injected into the MFB to induce a nearly complete loss of SNc DA neurons and a partial lesion of VTA DA neurons to model the advanced stage of parkinsonism. Animal surgeries and electrophysiology recordings were performed by different experimenters who were blinded to the treatments whenever possible. Parkinsonian phenotypes of 6-OHDA–injected mice were validated by (1) >80% loss of striatal TH immunoreactivity and (2) behavioral assessment: all mice were subject to (1) locomotion test in an open arena, where their locomotor activity and rotations were monitored for 10 min and quantified using ANYmaze software (Stoelting) and (2) cylinder test using a glass beaker (600 ml) to assess the use of the spontaneous forelimbs during weight-bearing touches, which were recorded using a digital camcorder at 60 fps (Panasonic, HC-V180 K) and analyzed off-line by an experimenter blinded to treatments.

##### Unsupervised clustering

To identify potential subtypes of PV^+^ cells in the present dataset, we conducted both *K*-means clustering and Ward's hierarchical clustering using Originlab Pro (2023). For electrophysiological parameters, *z*-scored data (i.e., variables are standardized with a mean of 0 and a standard deviation of 1) were used for unsupervised *K*-means clustering analysis and Ward's hierarchical clustering analysis. In *K*-means analysis, clusters of cells were generated to minimize within-cluster Euclidean distance. In Ward's hierarchical clustering analysis, a dendrogram was built using Euclidean distance to quantify the similarity between cells and clusters of cells.

##### Statistics analysis

Statistics were performed in GraphPad Prism 9 (GraphPad Software; RRID:SCR_002798). The nonparametric Mann–Whitney *U* (MWU) test was used to compare the medians of the two groups. A two-way analysis of variance (ANOVA) followed by Tukey's multiple-comparisons test was used to compare group differences when three or more groups/treatments were involved. Data were collected from three to eight mice per study. Individual animals were considered independent samples. All statistic tests were two-tailed with *p* values of <0.05 (*), 0.01 (**), or 0.001 (***) as thresholds for statistical significance. Data are reported as median plus interquartile ranges (in square brackets, Table 1).

## Results

Mice with 6-OHDA lesions of the MFB have been widely used as a model of nigrostriatal dopaminergic degeneration, and they also show certain levels of mesocortical dopaminergic degeneration (Swanson et al., 2021). In the present study, we quantified the tyrosine hydroxylase (TH) immunoreactivity (ir) in both the striatum and the M1. Mice with 6-OHDA lesions showed a nearly complete loss of striatal TH-ir (ipsilateral/contralateral striatal TH-ir%, controls = 93.5 [80.7, 109.3]%, *n* = 8 mice; 6-OHDA = 0 [0, 3.3]%, *n* = 9 mice; *p* < 0.0001, MWU), a moderate reduction of TH-ir in M1 (ipsilateral/contralateral cortical TH-ir%, controls = 100 [60, 170]%, *n* = 4 mice; DD = 35 [10, 51]%, *n* = 5 mice, *p* = 0.0017, MWU, Table 1), and a significant reduction of dopaminergic neurons in the ventral tegmental area (VTA, ipsilateral/contralateral TH-ir cells%, controls = 110 [82–130]%, *n* = 3 mice; DD = 43 [40, 47]%, *n* = 3 DD mice; *p* = 0.1, MWU, Table 1). These results are consistent with the literature showing that VTA neurons degenerate in this model (Swanson et al., 2021) and that dopaminergic inputs to the M1 mainly originate from the VTA in rodents (Hosp et al., 2011). Together, mice with 6-OHDA lesions of MFB induced nearly complete and moderate DA depletion in the dorsal striatum and M1, respectively. Thus, in the following sections, we will interrogate how the degeneration of midbrain dopaminergic neurons affects the GABAergic circuits formed by PV^+^ cells in M1.

### Loss of midbrain DA neurons does not change the number of PV^+^ cells in M1

**Figure 1.**
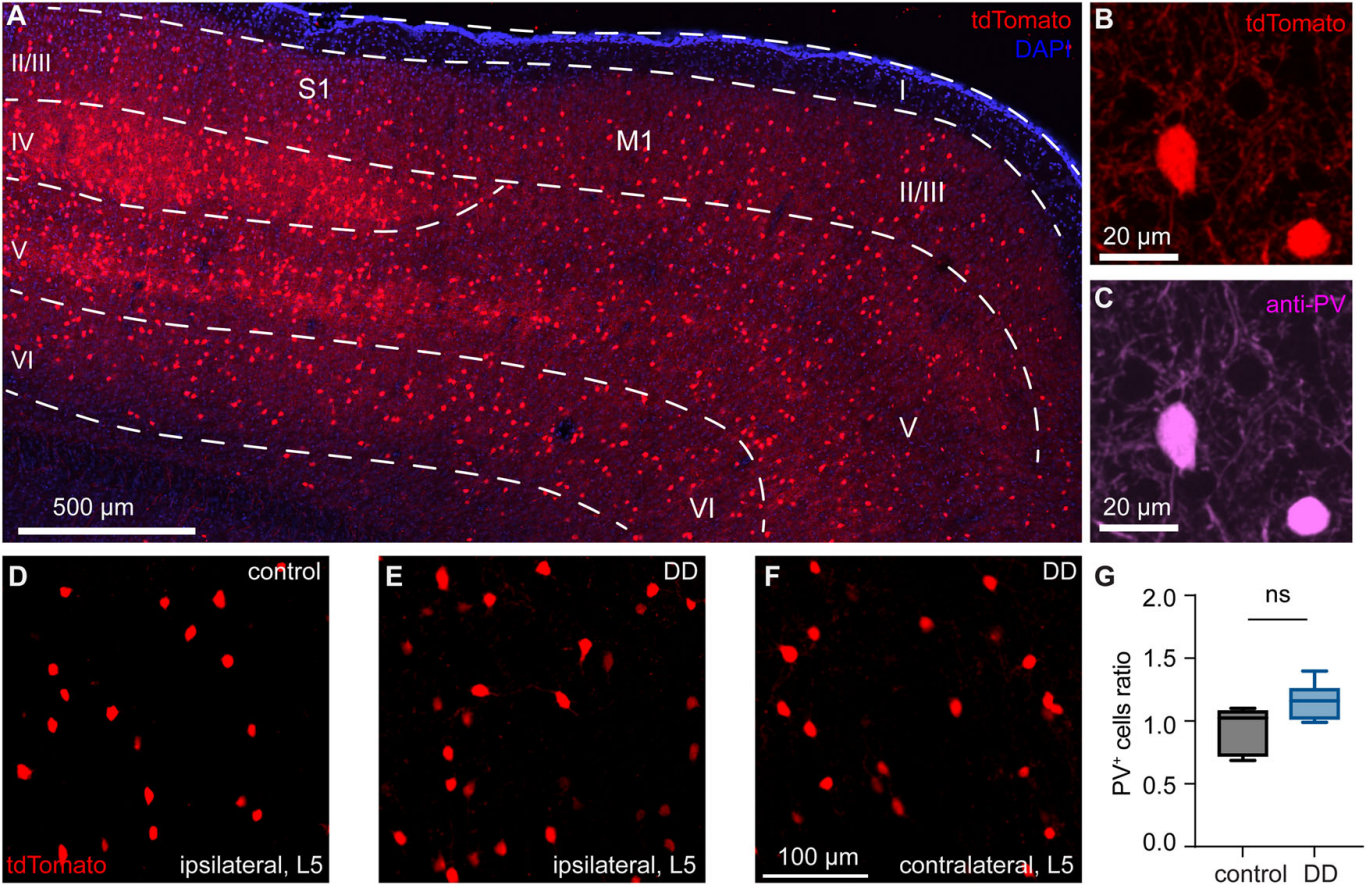
Loss of midbrain DA neurons does not change the number of PV^+^ cells in M1. ***A***, Representative confocal image of a sagittal section showing the distribution of tdTomato^+^ cells in the cerebral cortex of a PV-tdTomato mouse. ***B, C***, Representative images showing the overlap between tdTomato fluorescence (***B***) and PV immunoreactivity (***C***) in two neurons in Layer 5 of M1. ***D–F***, Representative confocal images showing PV^+^ cells in Layer 5 of M1 from the ipsilateral hemisphere of a control mouse (***D***) and both ipsilateral (***E***) and contralateral (***F***) hemispheres of a DA-depleted mouse. ***G***, The summarized results showing no difference in the densities of L5 PV^+^ cells between controls and DD mice (ratio of ipsi-/contralateral M1 PV^+^ cells, controls = 1.02 [0.73, 1.08], *n* = 5 mice; DD = 1.16 [1.02, 1.25], *n* = 6 mice; *p *= 0.08, MWU, Table 1). ns, not significant.

To study the effect of morphology and physiology of cortical PV^+^ cells, we crossed the PV-Cre knockin mice with the Ai14-tdTomato reporter line to express red fluorescent protein tdTomato in PV-expressing cells. In M1, PV^+^ cells could be found from Layer (L) 2/3 throughout L6 (Fig. 1*A*). The laminar distribution of PV^+^ cells is consistent with documented evidence in the literature (Rudy et al., 2011; Tremblay et al., 2016). Immunohistochemical staining showed that >95% tdTomato-expressing cells in M1 show PV immunoreactivity (PV-ir/tdTomato^+^ cells, 100 [83, 100]%, 11 slices/3 mice, Fig. 1*B,C*).

Next, we examined whether loss of DA alters the number of PV^+^ cells in M1. To do this, we employed an unbiased stereological approach to quantify the number of tdTomato^+^ neurons (i.e., PV^+^ cells) in L5 of the M1 from both DD mice and controls. We found no change in the number of L5 PV^+^ cells in DD mice relative to controls (Fig. 1*G*, Table 1). These data suggest no PV^+^ cell loss in M1 after the degeneration of midbrain dopaminergic neurons. This conclusion is consistent with an earlier study in human postmortem tissues showing an unaltered number of PV^+^ cells in PD patients (Lanoue et al., 2013).

### Loss of DA neurons does not change the morphology of PV^+^ cells in M1

**Figure 2.**
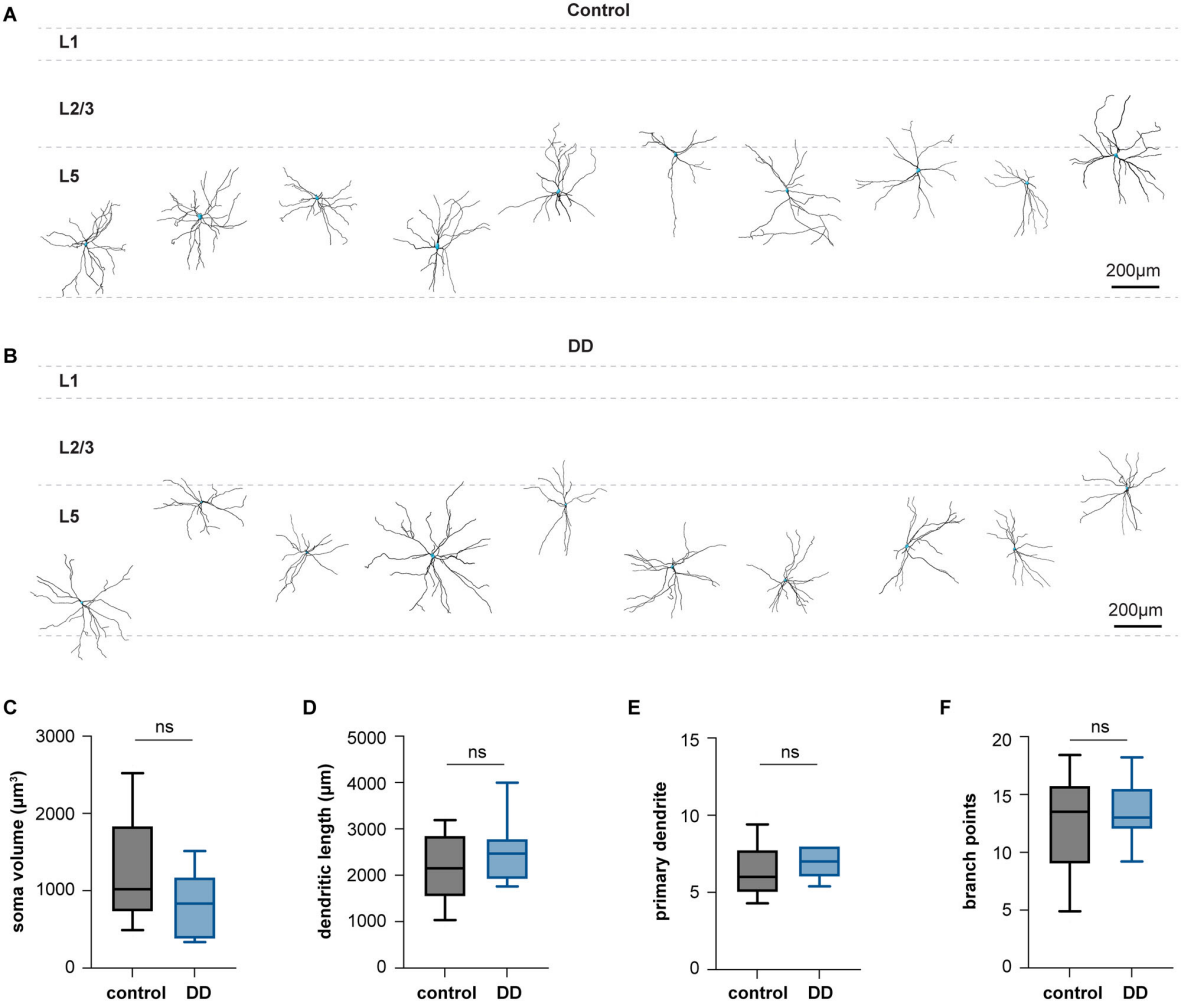
Loss of DA neurons induces subtle changes in the morphology of PV^+^ cells in M1. ***A, B***, Dendritic field of reconstructed L5 PV^+^ cells in the M1 of controls (***A***) and DD mice (***B***). ***C***, Box plot showing no difference in the soma volume of PV^+^ cells between controls and DD mice (control = 1,022 [728, 1,838] µm^3^, DD = 835 [376, 1,177] µm^3^, *p *= 0.09, MWU, Table 1). ***D***, Box plot showing no difference in the dendritic length of PV^+^ cells between controls and DD mice (control = 2,151 [1543, 2,856] µm, DD = 2,469 [1914, 2,786] µm, *p* = 0.27, MWU, Table 1). ***E***, Box plot showing no difference in the number of primary dendrites of PV^+^ cells between controls and DD mice (control = 6 [5, 7.8], DD = 7 [6, 8], *p* = 0.3, MWU, Table 1). ***F***, Box plot showing no difference in the number of branch points of PV^+^ cells between controls and DD mice (control = 13.5 [9, 15.75], DD = 13 [12, 15.5] µm, *p* > 0.9, MWU, Table 1). Morphology data were collected from and compared between 12 cells/3 mice for controls and 13 cells/3 mice for DD mice.

To assess the effect of DA depletion on the morphology, we labeled PV^+^ cells in Layer 5 of M1 with biocytin through whole-cell patch-clamp pipettes. The morphology of biocytin-filled neurons was revealed by Cy5-conjugated streptavidin. Following 3D reconstruction using Imaris software (Fig. 2*A,B*), the morphology of PV^+^ cells was compared between controls and DD mice. We found that there was no change in the major measure of neuronal morphology, including the volume of soma (Fig. 2*C*, Table 1), dendritic length (Fig. 2*D*, Table 1), number of primary dendrites (Fig. 2*E*, Table 1), and number of branch points (Fig. 2*F*, Table 1). The data suggest that loss of DA neurons did not change the morphology of PV^+^ cells in Layer 5 of the M1.

### Loss of dopaminergic neurons does not alter membrane properties of PV^+^ cells in M1

**Figure 3.**
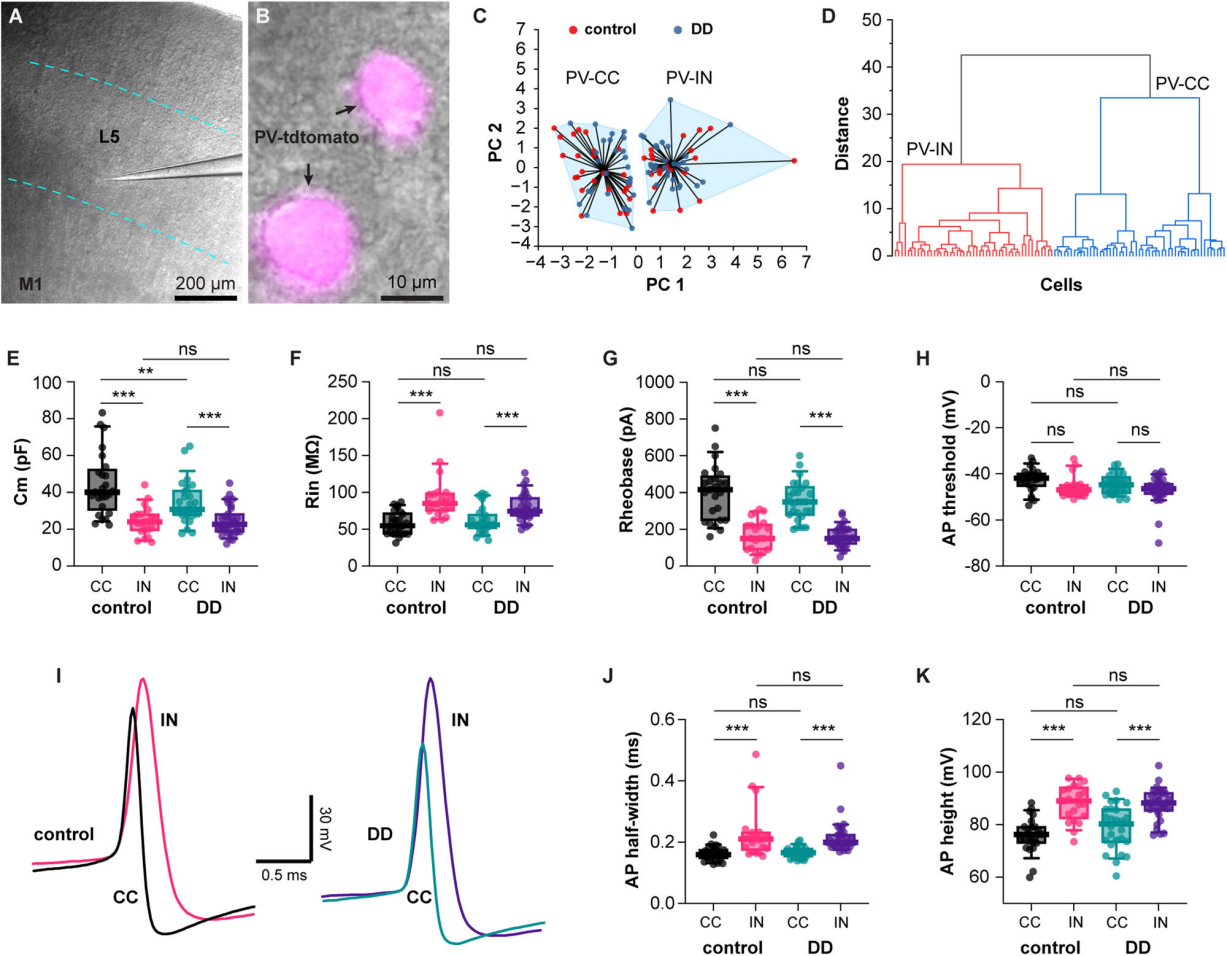
Loss of DA neurons does not alter the membrane properties of PV^+^ cells in M1. ***A, B***, Images showing the recording location of PV^+^ cells from Layer 5 of M1 (***A***) guided by the tdTomato fluorescence (***B***). ***C***, The *K*-means plot showing two separated clusters of PV^+^ cells in both controls and DD mice. ***D***, The dendrogram showing two clusters of PV^+^ cells identified by using Ward's hierarchical clustering in both controls and DD mice. ***E***, The summarized results showing differences in Cm of PV^+^ cells between clusters in both controls and DD mice (control/PV-CC = 40.1 [30.5, 52.4] pF, control/PV-IN = 24.1 [19.5, 28.2] pF, *p* < 0.0001; DD/PV-CC = 30.9 [27.1, 40.9] pF, DD/PV-IN = 22.7 [18.7, 28.3] pF, *p* = 0.003, Table 1). PV-CC cells in DD mice showed a decreased Cm relative to those in controls (*p* = 0.0082). ***F***, The summarized results showing differences in Rin of PV^+^ cells between clusters in both controls and DD mice (control/PV-CC = 54.8 [44, 72.0] MΩ, control/PV-IN = 85.2 [75.3, 98.6] MΩ, *p* < 0.0001; DD/PV-CC = 56.2 [51.4, 70.1] MΩ, DD/PV-IN = 74.7 [68.5, 92.7] MΩ, *p* < 0.01, Table 1). There was no difference in Rin of PV-CC or PV-IN cells between controls and DD mice. ***G, H***, Similar to ***F*** but for the rheobase (***G***, control/PV-CC = 415 [250, 488] pA, control/PV-IN = 151 [90, 228] pA, *p* < 0.0001; DD/PV-CC = 350 [278, 431] pA, DD/PV-IN = 150 [120, 200] pA, *p* < 0.0001, Table 1) and AP threshold (***H***, controls/PV-CC = −41.9 [−45.4, −39.9] mV, control/PV-IN = −47 [−49.1, −44.8] mV, *p* = 0.125; DD/PV-CC = −44.8 [−48.3, −41.3] mV, DD/PV-IN = −46.5 [−48.7, −44.3] mV, *p *= 0.2, Table 1). ***I***, Representative traces of APs from PV-CC and PV-IN cells from controls (left) and DD mice (right). ***J***, Similar to ***F*** but for the AP half-width (control /PV-CC = 0.16 [0.15, 0.18] ms, control/PV-IN = 0.21 [0.17, 0.23] ms, *p* = 0.0003; DD/PV-CC = 0.17 [0.15, 0.18] ms, DD/PV-IN = 0.20 [0.19, 0.23] ms, *p* < 0.003, Table 1). ***K***, Similar to ***F*** but for the AP height (control/PV-CC = 76.3 [73.0, 79.2] mV, control/PV-IN = 89.1 [82.4, 94.2] mV, *p* < 0.0001; DD/PV-CC = 80.3 [73.3, 85.9] mV, DD/PV-IN = 88.3 [85.2, 92.1] mV, *p* < 0.0001, Table 1). Statistics were conducted based on 25 PV-CC and 21 PV-IN cells from seven controls, as well as 26 PV-CC and 31 PV-IN cells from eight DD mice using two-way ANOVA followed by Tukey's test.

Next, we performed whole-cell current-clamp recording to assess the membrane properties of PV^+^ cells from controls and DA-depleted PV-tdTomato mice (Fig. 3*A,B*). Heterogeneity in PV^+^ cells with different morphological and physiological features has been documented in the neocortex (Naka and Adesnik, 2016; Tremblay et al., 2016). Particularly, recent work reported that Layer 5 long-range projecting PV^+^ cells connecting two hemispheres via the corpus callosum (PV-CC) are distinct from PV^+^ fast-spiking interneurons (PV-INs) in the M1 and other cortical subregions (Rock et al., 2018; Zurita et al., 2018). Thus, we tested if the putative PV-CC cells in Layer 5 of M1 can be distinguished from the PV-INs based on basic electrophysiological parameters.

To do this, the following measures represented the subthreshold, and suprathreshold membrane properties were chosen for unsupervised clustering analysis, including the cell capacitance (Cm), membrane input resistance (Rin), resting membrane potential (RMP), rheobase current, and the threshold, width, and height of action potential (AP) at rheobase. We then normalized the data and conducted an unsupervised *K*-means clustering algorithm to classify PV^+^ cells from both controls and DA-depleted mice. The *K*-means clustering algorithm generated two distinct clusters of PV^+^ cells (Fig. 3*C*). Mapping the cellular identities back to the *K*-means clusters revealed that the cells in two clusters correspond to the putative PV-CC and PV-IN subtypes, which was supported by their significantly different physiological properties (Fig. 3*E–K*) as outlined below. Moreover, each cluster comprised cells from both controls and DD mice, that is, cells from different treatment groups were not separated by this clustering algorithm. Additionally, unsupervised hierarchical clustering analysis also generated two main clusters of PV^+^ cells, which can be mapped as putative PV-CC and PV-IN subtypes, respectively, based on their physiological properties (Fig. 3*D*). Compared with the clusters from *K*-means analysis, only three cells showed switched membership in the hierarchical clustering analysis. Thus, we presented the quantification and comparison of electrophysiological parameters of two clusters from the *K*-means clustering analysis below.

In the control group, 54.4 and 45.6% PV^+^ cells were identified as putative PV-CC and PV-INs, respectively. Similarly, in DD mice, 46.4 and 53.6% of PV^+^ cells were categorized as putative PV-CC and PV-INs, respectively. No difference was observed in the proportion of PV-IN cells between controls and DD mice (*p* = 0.4, chi-squared test).

The putative PV-CC and PV-IN cells are different across several membrane properties. Specifically, PV-CC cells show larger Cm (Fig. 3*E*) and smaller Rin (Fig. 3*F*) than PV-INs in both controls and DD mice. Regarding cellular excitability, PV-CC exhibit higher rheobase currents but comparable AP threshold relative to PV-INs (Fig. 3*G,H*). When it comes to AP morphology, PV-CC cells exhibited narrower APs (Fig. 3*I,J*) and lower APs in height (Fig. 3*I,K*) relative to PV-INs in both controls and DD mice. Together, the distinct electrophysiological properties support the conclusion that PV^+^ cells in controls and DD mice include both putative PV-CC and PV-IN subtypes (Rock et al., 2018; Zurita et al., 2018).

Next, we compared the membrane properties of PV^+^ cell subtypes between controls and DD mice to assess the impact of DA depletion on the cellular excitability of PV-INs in M1. For the putative PV-CC cells, we detected a significant reduction of Cm between controls and DD mice (Fig. 3*E*). There was no change in the other parameters of PV-CC cells between controls and DD mice, including the Rin (Fig. 3*F*), the rheobase (Fig. 3*G*), the threshold (Fig. 3*H*), the half-width (Fig. 3*J*), and the AP height (Fig. 3*K*). For PV-INs, there was no difference in all membrane properties measured between controls and DD mice (Fig. 3*E–K*). The above results suggest that the loss of midbrain DA neurons does not change the basic membrane properties of PV^+^ cells in M1.

**Figure 4.**
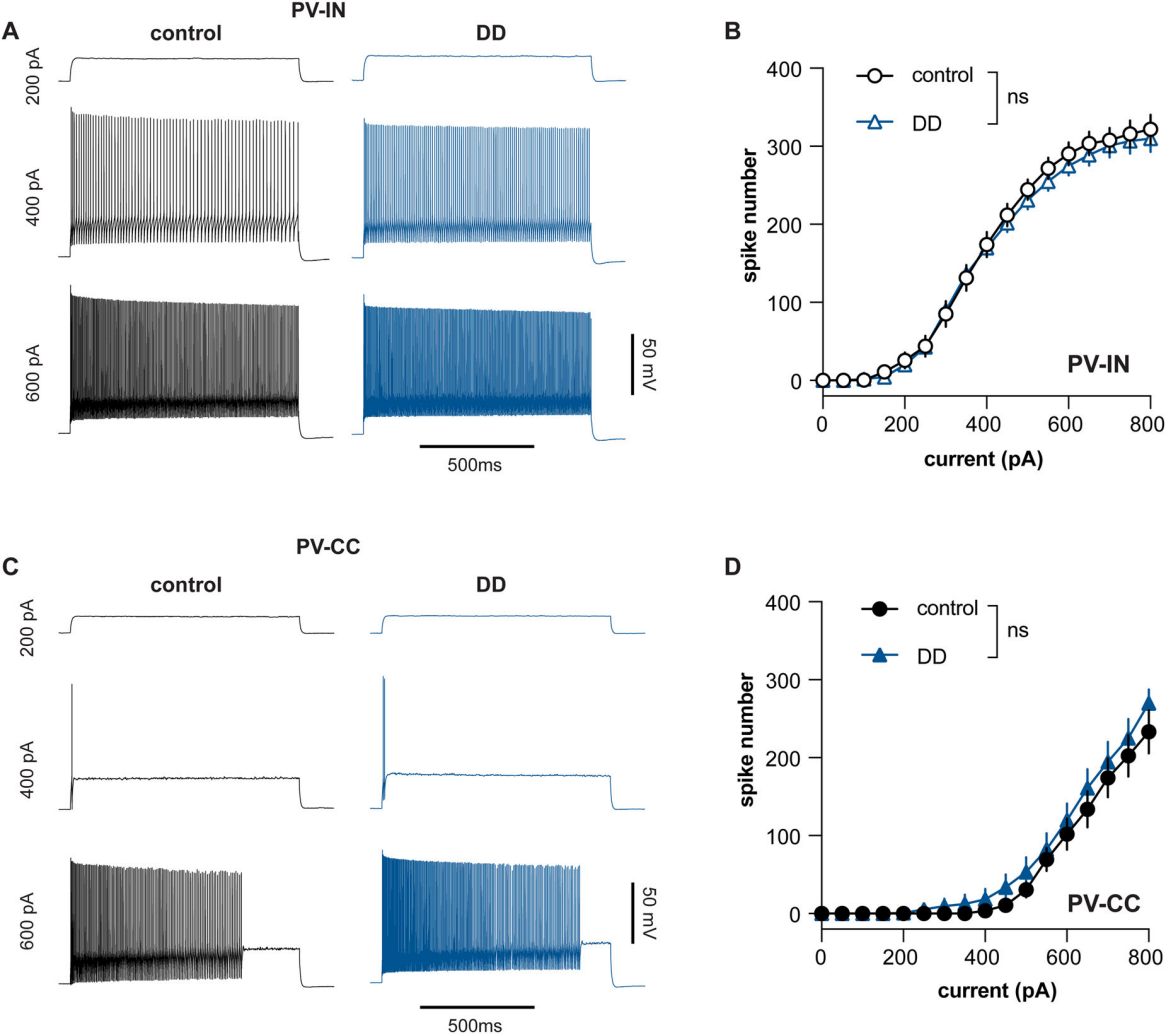
Loss of DA does not change the intrinsic excitability of PV^+^ cells in L5 of M1. ***A***, Representative trains of spikes evoked by different levels of somatic current injections into the PV-IN cells of controls and DD mice. ***B***, F–I curve of L5 PV-INs in M1 from controls and DD mice. ***C, D***, Similar to ***A*** and ***B*** but from PV-CC cells. Group difference *p* > 0.05 for curves in ***B*** and ***D***, two-way ANOVA, Table 1.

To further study the impact of DA depletion on the excitability of PV^+^ cells, we injected a family of depolarizing current (1 s) to evoke trains of spikes and compare the profiles of frequency–current (F–I) curves of PV^+^ cell subtypes between groups. In both controls and DD mice, the F–I curve of PV-INs was relatively left-shifted compared with that of the putative PV-CC cells (Fig. 4*B,D*), which is consistent with their lower rheobase currents (Fig. 3*G*). Moreover, PV-INs exhibited typical fast-spiking firing properties in response to a large amount of somatic current injection (Fig. 4*A,B*). The putative PV-CC cells often showed a stuttering pattern of firing and ceased firing toward the end of 1 s current injection (Fig. 4*C*). Eventually, the putative PV-CC cells also could discharge at high frequency in response to a large amount of somatic current injection (Fig. 4*C,D*). However, there was no change in the overall profiles of F–I curves of PV-CC (Fig. 4*B*) or PV-IN (Fig. 4*D*) cells between controls and DD mice. Together, the above result suggests that the loss of SNc DA neurons does not induce changes in the subthreshold and suprathreshold membrane properties of PV^+^ cells in M1.

### Loss of DA does not alter GABAergic innervation of M1 pyramidal neurons

PV^+^ cells play an important role in gating the excitability of cortical pyramidal neurons and contribute to cortical circuit computation through robust perisomatic GABAergic inhibition. Next, we studied the effects of loss of midbrain DA neurons on the connection of GABAergic projections from PV^+^ cells to cortical pyramidal neurons. We recently reported that loss of midbrain DA neurons induces cell-subtype- and input-selective changes in neuronal excitability and synaptic strength in M1 circuits (Chen et al., 2021, 2023). Thus, we employed immunohistochemistry, electrophysiology, retrograde tracing, and optogenetics to study the connectivity of GABAergic synapses between the PV^+^ cells and retrogradely labeled M1 PT and IT neurons in both controls and DD mice.

**Figure 5.**
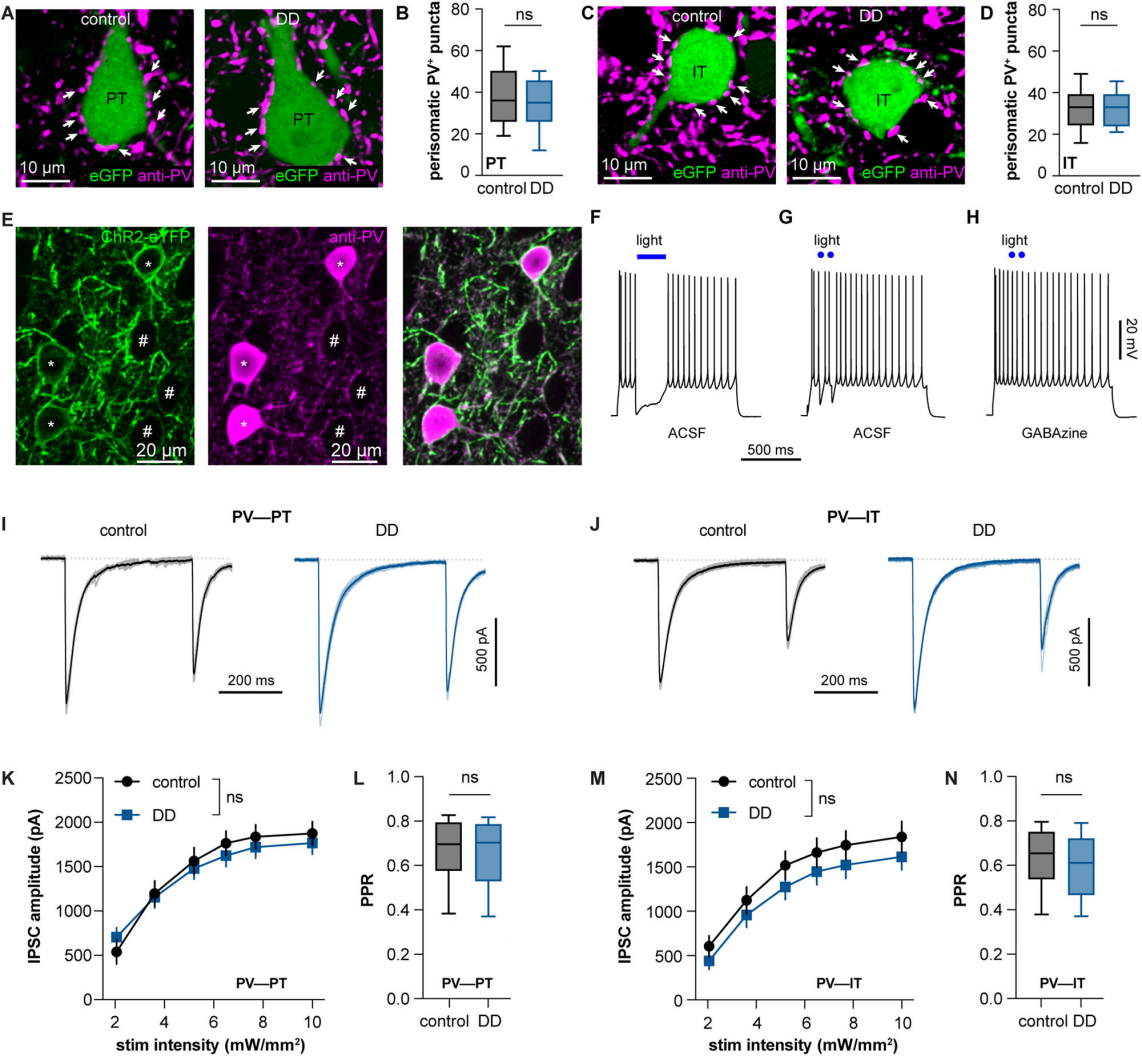
Loss of DA does not alter GABAergic innervation of M1 pyramidal neurons. ***A***, Representative confocal image showing PV^+^ perisomatic puncta (arrows) around eGFP-labeled PT neurons from controls and DD mice. ***B***, Box plot showing no change in the number of perisomatic PV^+^ puncta around PT neurons between controls and DD mice (control = 36 [26, 50], *n* = 39 cells/4 mice; DD = 35 [26, 46], *n* = 45 cells/4 mice; *p* = 0.28, MWU, Table 1). ***C, D***, Similar to ***A*** and ***B*** but for IT neurons (control = 33 [25, 39], *n* = 37 cells/4 mice; 6-OHDA = 33 [24, 39], *n* = 44 cells/4 mice; *p* = 0.8, MWU, Table 1). ***E***, Representative images showing ChR2-eYFP–expressing terminals (left) were PV immunoreactive (middle and right) in the M1 of PV-ChR2 mice. * highlights PV^+^ cells with ChR2-eYFP expression on the membrane surface. # highlights putative cortical pyramidal neurons surrounded by PV^+^ perisomatic puncta. ***F–H***, Representative spike traces of M1 pyramidal neurons of PV-ChR2 mice and their responses to long (blue bar) and short (blue dots) optogenetic stimulation (i.e., blue light) in regular ACSF and ACSF with GABA_A_ receptor antagonist GABAzine (10 µM). ***I***, Representative traces of optogenetically evoked IPSCs in PT neurons from controls and DD mice. Optogenetic stimulation intensity, 3.6 mW/mm^2^. ***K***, Stimulation–amplitude curve of PV IPSCs in PT neurons from controls (*n* = 33 cells/4 mice) and DD mice (51 cells/8 mice). *p* > 0.05 for group difference, two-way ANOVA, Table 1. ***L***, Box plot showing no changes in the paired-pulse ratio (PPR) of IPSCs in PT neurons between controls and DD mice (control = 0.7 [0.58, 0.79], *n* = 33 cells/4 mice; DD = 0.7 [0.53, 0.79], *n *= 51 cells/8 mice, *p* = 0.9, MWU, Table 1). ***J, M, N***, Similar to ***I***, ***K***, and ***L*** but for M1 IT neurons (PPR in ***N***, control = 0.65 [0.54, 0.75], *n *= 32 cells/4 mice, DD = 0.61 [0.47, 0.72], *n *= 33 cells/5 mice, *p *= 0.23, MWU, Table 1).

PT and IT neurons were retrogradely labeled by injecting AAVrg-hsyn-eGFP into the pontine nuclei and contralateral striatum, respectively, as described previously (Chen et al., 2021, 2023). PV immunoreactive perisomatic puncta around the eGFP-labeled PT and IT neurons from controls and 6-OHDA mice were quantified (Fig. 5*A,C*). The number of PV^+^ puncta from PT neurons was not altered by loss of DA (Fig. 5*A,B*). Moreover, the number of PV^+^ puncta from IT neurons was not altered either by the loss of DA (Fig. 5*C,D*). In addition, there was no difference in the number of PV^+^ perisomatic puncta between PT and IT neurons from controls, indicating that PT and IT neurons in M1 receive comparable levels of perisomatic GABAergic inhibition from PV^+^ cells. This is consistent with the dense but unspecific innervation of cortical pyramidal neurons by PV^+^ interneurons in the neocortex (Packer and Yuste, 2011). These data indicate that the number of perisomatic GABAergic synapses to PT and IT neurons is not altered by the loss of DA.

Next, we compared the GABAergic synaptic connection strength between PV^+^ cells and PT or IT neurons in both controls and DD mice. To do this, we induced midbrain DA neurodegeneration in PV-ChR2 knockin mice, allowing us to selectively activate and assess GABAergic synaptic transmission arising from the PV^+^ cells (Fig. 5*E*). Consistent with the robust perisomatic innervation, activation of ChR2 expressing PV^+^ axon terminals could stop cortical pyramidal neuronal firing (Fig. 5*F,G*), an effect that could be abolished by GABA_A_ receptor antagonist (Fig. 5*H*). Furthermore, we quantified the connection strength of GABAergic synapses between PV^+^ cells and M1 pyramidal neuron subtypes. To do this, we recorded and assessed optogenetically evoked IPSCs from retrogradely labeled PT and IT neurons in M1 under the whole-cell voltage-clamp mode. There was no difference in the amplitude of optogenetically evoked IPSCs in PT neurons between controls and DD mice (Fig. 5*I,K*). Similarly, there was no change in the amplitude of optogenetically evoked IPSCs in IT neurons between control and DD mice (Fig. 5*J,M*). In addition, there was also no change in the ratios of the amplitude of IPSCs evoked by paired optogenetic stimulations in either PT (Fig. 5*L*) or IT (Fig. 5*N*) neurons between controls and DD mice. These data suggest that there was no change in the initial release probability of GABA at PV-expressing presynaptic axon terminals. Together, both the anatomical evidence and physiology data suggest that loss of DA does not affect the inhibitory innervation of cortical pyramidal neurons by PV^+^ cells.

## Discussion

Pyramidal neurons in the motor cortex exhibit a highly synchronized bursting pattern of activity in the parkinsonian state that can be suppressed by deep brain stimulation, indicating that it is pathological in nature (Goldberg et al., 2002; Pasquereau and Turner, 2011; Li et al., 2012). Considering the well-established role of PV^+^ cells in controlling the timing and magnitude of cortical pyramidal neuronal activity, the present study examined potential changes of the PV^+^ cells following the loss of midbrain DA neurons in mice to provide insight into the cellular and synaptic mechanisms of the pathological pattern of cortical activity in the parkinsonian state. We documented that neither the morphology nor the physiology of M1 PV^+^ cells was altered following the degeneration of midbrain DA neurons. Furthermore, the GABAergic connection between the PV^+^ cells and M1 pyramidal neuron subtypes was not altered by the loss of midbrain DA neurons. Together, this study demonstrated that the activity of PV^+^ cells and their GABAergic control of M1 pyramidal neurons are intact in the parkinsonian state.

### Cortical GABAergic network dysfunction in PD

A large body of evidence suggests that the pathophysiology of motor symptoms in PD involves cortical GABAergic mechanisms. Studies in postmortem human brains of people with PD reported a significant reduction of glutamic acid decarboxylase 67 (GAD67), a critical enzyme for GABA synthesis, in the prefrontal cortex (Lanoue et al., 2010). Moreover, upregulation of GABA_A_ receptor function using zolpidem could concurrently suppress the β-band activity in M1 and bradykinesia of PD patients (Prokic et al., 2019). Although the exact mechanisms of zolpidem's therapeutic effects remain undefined, it indicates that the generation of the β-band activity in M1 involves GABA_A_ receptors in either cortical or subcortical regions or both. In animal model studies, M1 interneurons show an increased firing frequency that is phase-locked to the cortical β-band activity (25–40 Hz) in parkinsonian rats (Brazhnik et al., 2012). Recent studies suggest that deep brain stimulation of the subthalamic nucleus could effectively decrease the synchronized bursting pattern of activity in M1 and alleviate parkinsonian motor deficits of DA-depleted mice, perhaps partially through recruiting cortical GABAergic interneurons (Li et al., 2012; Valverde et al., 2020). Together, compelling evidence from both clinical and preclinical studies strongly suggests a key role of M1 GABAergic interneurons in the pathophysiology and therapeutic alleviation of motor symptoms in parkinsonism.

Building on the above studies, we conducted detailed analyses of the inhibitory network formed by PV^+^ cells after the loss of midbrain DA neurons. We found that the number of PV^+^ cells in M1 was not altered in DA-depleted mice (Fig. 1). This conclusion is consistent with an earlier human study showing that the number of PV^+^ cells was not altered in the prefrontal cortex of postmortem brains from PD patients (Lanoue et al., 2013). The same study by Lanoue et al (2013) also reported a significant reduction of parvalbumin mRNA expression at the cellular level in PD cases. Considering that as a Ca^2+^ buffering protein parvalbumin plays a key role in regulating intracellular Ca^2+^ concentration, a decreased PV expression level may affect the physiological properties and synaptic dynamics of cortical PV^+^ cells (Eggermann and Jonas, 2012). Our results showed that the overall morphology (Fig. 2), the cellular physiology (Figs. 3, 4), and the synaptic physiology (Fig. 5) of Layer 5 PV^+^ cells were not altered by the loss of midbrain DA neurons. It is worth noting that dopaminergic projections to M1 mainly originate from the VTA in rodents, which was also partially lesioned in mice with 6-OHDA injection into the MFB. Although PV^+^ cells are targeted by dopaminergic inputs, a partial loss of VTA dopaminergic neurons seems not sufficient to trigger significant changes in PV^+^ microcircuits in M1. Together, our results from anatomical and physiological studies suggest an intact GABAergic network formed by PV^+^ cells in M1 in the parkinsonian state.

Layer 5 PV^+^ cells in M1 are primarily targeted by thalamic inputs in the physiology state (Tremblay et al., 2016; Okoro et al., 2022). Thus, the PV^+^ cells and their perisomatic connection with M1 pyramidal neurons are readily recruited by synchronized thalamic inputs in parkinsonism (Nakamura et al., 2021), contributing to the generation of the abnormal pattern of pyramidal neuronal firing in the parkinsonian state.

### Heterogeneity of PV^+^ cells in the M1

The heterogeneity of neocortical PV^+^ cells based on morphological features (e.g., basket vs chandelier cells) has been documented (Tremblay et al., 2016). The PV^+^ cells in the present study were mainly from Layer 5 of M1 and they were likely the fast-spiking basket interneurons. Of relevance to the present work, PV-CC cells account for ∼40% of all PV^+^ cells in various subregions of the neocortex and send long-range projections to the contralateral hemisphere via the corpus callosum (Rock et al., 2018). Like the PV^+^ interneurons, PV-CC cells also directly innervate cortical pyramidal neurons through monosynaptic and perisomatic GABAergic synapses and receive monosynaptic excitation from the thalamus (Rock et al., 2018). PV-CC cells share morphological features with PV-IN cells in the cerebral cortex, including a cross-layer dendritic coverage, allowing them to receive different afferents. Relative to the PV-INs, PV-CC cells exhibit narrower AP width, delayed AP firing, and higher rheobase, as well as a non–fast-spiking pattern of repetitive firing (Zurita et al., 2018). The expression of Kv1.1 conductance has been linked with the unique pattern of firing of PV-CC cells. Building on these documented morphological and physiological differences, we proposed that the two clusters of PV^+^ cells in our dataset that were identified by unsupervised clustering algorithms belong to PV-CC and PV-INs, respectively. Interestingly, we also found a subtle but significant change in the soma size of PV-CC cells after DA depletion from electrophysiology properties (Fig. 3*E*). These results indicate that PV-CC cells may exhibit subtle adaptative changes in response to loss of DA in the parkinsonian state, although the significance of such changes remains to be determined.

The main conclusion of the present study is consistent with a recent work using the same mouse model of hemiparkinsonism (Swanson et al., 2023). Although very subtle changes in the intrinsic excitability of and thalamic inputs to Layers 2/3 PV^+^ cells were reported in this work, there were no robust phenotypes documented for Layer 5 PV^+^ cells of parkinsonian mice. Of worth noting, PV^+^ cells in M1 express DA D2 receptors and are the major targets of dopaminergic inputs arising from the VTA (Cousineau et al., 2020). The M1 of primates receives far more intense dopaminergic inputs from both the VTA and the SNc compared with that in rodents (Williams and Goldman-Rakic, 1998; Hosp et al., 2011). Thus, future studies remain needed to determine the potential impact of a severe loss of DA neuromodulation on PV^+^ microcircuits and its possible contributions to the pathophysiology of motor deficits in human PD (Guo et al., 2015).

Last, the present study provides evidence showing an intact perisomatic inhibition of cortical pyramidal neurons by PV^+^ cells in the parkinsonian state. It is worth noting that the PV-ChR2 mice did not allow us to separate and compare the inhibition of pyramidal neurons by PV-CC or PV-INs between controls and DD mice. Moreover, systematic experimental and computational approaches are needed to examine potential changes in the other subclasses of GABAergic interneurons in M1 to better understand the role of cortical GABAergic network in shaping the rate and pattern of pyramidal neuronal firing in a cell-subtype–specific manner in PD (Goldberg et al., 2002; Pasquereau and Turner, 2011).

## Synthesis

Reviewing Editor: Laura Volpicelli-Daley, The University of Alabama at Birmingham Decisions are customarily a result of the Reviewing Editor and the peer reviewers coming together and discussing their recommendations until a consensus is reached. When revisions are invited, a fact-based synthesis statement explaining their decision and outlining what is needed to prepare a revision will be listed below. The following reviewer(s) agreed to reveal their identity: NONE. Note: If this manuscript was transferred from JNeurosci and a decision was made to accept the manuscript without peer review, a brief statement to this effect will instead be what is listed below.

Overall, the manuscript is missing two key pieces of data, 1) stereological counts of PV interneurons in M1 and 2) quantitation of TH in M1 and the VTA on at least 3 sections from all lesioned and non-lesions mice.

Specific Reviewer's comments:

There is a recent report from the Maffei Lab that reports the effects of dopamine depletion on pyramidal cells and PV+ cells in M1 both in layer 2/3 and layer 5. The published study mostly examines excitatory input into M1 from the thalamus. However, this study directly examines layer 5 PV in a much more comprehensive and focused manner.

This paper thoroughly examines how SNc dopamine depletion effects M1 PV+ cells and how this could possibly relate to the well charecterized synchronization of bursting activity in parkinsonian states. The authors use multiple immunohistochemical and elecrophysilogical measures to charecterize M1 PV+ cells, and additionally break them out into cells that putatively cross the corpus callosum or local interneurons. Largely I do not have any experimental or methodological concerns. The one exception to this is how the counting of PV+ cells was performed. Using more robust stereological methods rather than counting a single defined section, would aid confidence in this result.

My major concern over these findings is conceptual. The authors examine striatal TH+ immunoreactivity and also quantify behavioral measures of dopamine lesioning to confirm 6-OHDA lesions. While this data is not presented, it would suggest that all animals used had a severe unilateral dopamine lesion, and that all recordings where thus done in mice with severe SNc cell loss. However, in mice, the VTA provides the major dopamine innervation to M1, and MFB lesions are not always as highly efficient at removing VTA dopamine neurons as in the SNc. Quantification of TH immunoreactivity in M1 would be inoformative of whether PV+ cells in this region were direcly receiving decreased dopaminergic signaling or these lack of changes were result of system effects rather than local dopaminergic effects. This would also change the interpretation of the lack of effect, and the only signigificant electrophysiological change noted on Cm of PV-CC cells may point to this.
